# Autoantibodies from patients with kidney allograft vasculopathy stimulate a proinflammatory switch in endothelial cells and monocytes mediated via GPCR-directed PAR1-TNF-α signaling

**DOI:** 10.3389/fimmu.2023.1289744

**Published:** 2023-10-30

**Authors:** Guido Moll, Christian Luecht, Michael Adu Gyamfi, Dennyson L. M. da Fonseca, Pinchao Wang, Hongfan Zhao, Zexian Gong, Lei Chen, Muhamad Imtiaz Ashraf, Harald Heidecke, Alexander Maximilian Hackel, Duska Dragun, Klemens Budde, Olaf Penack, Gabriela Riemekasten, Otávio Cabral-Marques, Janusz Witowski, Rusan Catar

**Affiliations:** ^1^ Department of Nephrology and Internal Intensive Care Medicine, Charité Universitätsmedizin Berlin, corporate member of Freie Universität Berlin, Humboldt-Universität zu Berlin, and Berlin Institute of Healthy (BIH), Berlin, Germany; ^2^ Berlin Institute of Healthy (BIH) Center for Regenerative Therapies (BCRT) and Berlin-Brandenburg School for Regenerative Therapies (BSRT), Charité Universitätsmedizin Berlin, Berlin, Germany; ^3^ Interunit Postgraduate Program on Bioinformatics, Institute of Mathematics and Statistics (IME), University of São Paulo (USP), São Paulo, Brazil; ^4^ Department of Surgery, Charité Universitätsmedizin Berlin, Berlin, Germany; ^5^ CellTrend GmbH, Luckenwalde, Germany; ^6^ Department of Rheumatology and Clinical Immunology, University of Lübeck, Lübeck, Germany; ^7^ Department of Hematology, Oncology and Tumorimmunology, Charité Universitätsmedizin Berlin, Berlin, Germany; ^8^ Berlin Institute of Health (BIH), Berlin, Germany; ^9^ Department of Clinical and Toxicological Analyses, School of Pharmaceutical Sciences, USP, São Paulo, Brazil; ^10^ Department of Medicine, Division of Molecular Medicine, USP School of Medicine, São Paulo, Brazil; ^11^ Laboratory of Medical Investigation 29, USP School of Medicine, São Paulo, Brazil; ^12^ Department of Immunology, Institute of Biomedical Sciences, USP, São Paulo, Brazil; ^13^ Department of Pathophysiology, Poznan University of Medical Sciences, Poznan, Poland

**Keywords:** chronic kidney disease (CKD), end-stage renal disease (ESRD), kidney transplantation (KTx), kidney allograft vasculopathy, endothelial cells (ECs), non-HLA-directed regulatory autoantibodies (RABs), autoantibodies, tumor necrosis factor-alpha (TNF-α)

## Abstract

Non-HLA-directed regulatory autoantibodies (RABs) are known to target G-protein coupled receptors (GPCRs) and thereby contribute to kidney transplant vasculopathy and failure. However, the detailed underlying signaling mechanisms in human microvascular endothelial cells (HMECs) and immune cells need to be clarified in more detail. In this study, we compared the immune stimulatory effects and concomitant intracellular and extracellular signaling mechanisms of immunoglobulin G (IgG)-fractions from kidney transplant patients with allograft vasculopathy (KTx-IgG), to that from patients without vasculopathy, or matched healthy controls (Con-IgG). We found that KTx-IgG from patients with vasculopathy, but not KTx-IgG from patients without vasculopathy or Con-IgG, elicits HMEC activation and subsequent upregulation and secretion of tumor necrosis factor alpha (TNF-α) from HMECs, which was amplified in the presence of the protease-activated thrombin receptor 1 (PAR1) activator thrombin, but could be omitted by selectively blocking the PAR1 receptor. The amount and activity of the TNF-α secreted by HMECs stimulated with KTx-IgG from patients with vasculopathy was sufficient to induce subsequent THP-1 monocytic cell activation. Furthermore, AP-1/c-FOS, was identified as crucial transcription factor complex controlling the KTx-IgG-induced endothelial TNF-α synthesis, and mircoRNA-let-7f-5p as a regulatory element in modulating the underlying signaling cascade. In conclusion, exposure of HMECs to KTx-IgG from patients with allograft vasculopathy, but not KTx-IgG from patients without vasculopathy or healthy Con-IgG, triggers signaling through the PAR1-AP-1/c-FOS-miRNA-let7-axis, to control TNF-α gene transcription and TNF-α-induced monocyte activation. These observations offer a greater mechanistic understanding of endothelial cells and subsequent immune cell activation in the clinical setting of transplant vasculopathy that can eventually lead to transplant failure, irrespective of alloantigen-directed responses.

## 1 Introduction

Over the past twenty years, non-HLA-directed regulatory autoantibodies (RABs) with the ability to target G protein-coupled receptors (GPCRs) have evolved as an essential new element in clinical pharmacology and multifaceted clinical pathology, e.g., in the setting of solid organ transplantation (SOT), cardiovascular diseases (CVDs), and in particular for various types of vasculopathy (1–3). This article aims to contribute to a better understanding of the molecular and cellular innate immune crosstalk that RABs can induce in the setting of transplant vasculopathy, which can lead to transplant failure (1).

Initial hallmark studies by Dragun et al. established the crucial functional role of immunoglobulin G (IgG)-derived RABs for the non-HLA-related outcome in kidney transplant (KTx) allograft survival in patients with allograft vasculopathy (4–8). Here, anti-GPCR-directed RABs associated with allograft vasculopathy constitute a distinct phenotype of antibody-mediated rejection (ABMR) (9, 10). After establishing the functional role of RABs directed against the angiotensin II type 1 receptor (AT1R) and endothelin receptor type A (ETAR) for vascular integrity and graft function/survival in the KTx setting (4, 8, 9, 11–16), subsequent studies have identified several RABs directed against GPCR targets typically associated with the vasculature, such as protease-activated receptors (PARs), neuronal and chemokine receptors, and the levels of these RABs are often modulated with patient age and disease status (1, 17, 18). Vasculature-associated RAB targets can be found on different types of micro-/macro-vascular endothelial cells (ECs), vascular smooth muscle cells (VSCMs), and perivascular multipotent mesenchymal stromal/stem cells (MSCs), but also neuronal cells, which are all often employed as model systems to study respective molecular signaling mechanisms (1, 19, 20).

It is now vital to decipher the detailed underlying signaling mechanisms, how these GPCR-specific functional RABs contribute to vascular dysfunction/injury, and the concomitant (auto)immune pathology in patients (1, 21, 22). Recently, we have demonstrated that serum-derived IgG fractions from patients with scleroderma-associated renal crisis stimulate interleukin-6 (IL-6) production by targeting the protease-activated receptor 1 (PAR1) on ECs (23). Intriguingly, histological features of systemic sclerosis (SSc) patients closely resemble those found in KTx recipients without detectable anti-HLA-antibodies but with features of chronic graft rejection (24). The concomitant vascular changes observed in these clinical settings include chronic inflammation, intimal fibrosis, and tissue calcification (1, 21, 22, 25). This pathology resembling a phenotype of chronic rejection must be distinguished from the more common phenotype and risk of acute rejection in the solid organ transplant (SOT) setting, which still accounts for the majority of graft rejections/failures, although overall 1-year allograft survival is now typically >90% (26–28).

A key mediator in (auto)immune pathology is the prototypic proinflammatory cytokine tumor necrosis factor alpha (TNF-α), typically released by activated leukocytes. Its production, underlying signaling mechanisms, and multiple biological effects have been studied extensively over the past three decades (29–32). Typically, ECs are considered to be targets for TNF-α. While TNF receptor 1 (TNFR1) is constitutively expressed on many cell types, expression of TNFR2 is restricted primarily to ECs (31, 32). When acting directly on ECs, TNF-α stimulates the expression of chemokines, adhesion molecules, and metabolites, such as eicosanoids, that are key to the effective inflammatory response (33). The effect of TNF-α on ECs has been extensively studied in the past (34), but the role of ECs as a significant source of TNF-α has only been tested rather sporadically so far, e.g., TNF-α secretion has been previously reported to occur in human umbilical vein ECs (HUVECs) stimulated with IL-1 and other proinflammatory stimuli (35, 36).

**Figure 1:**
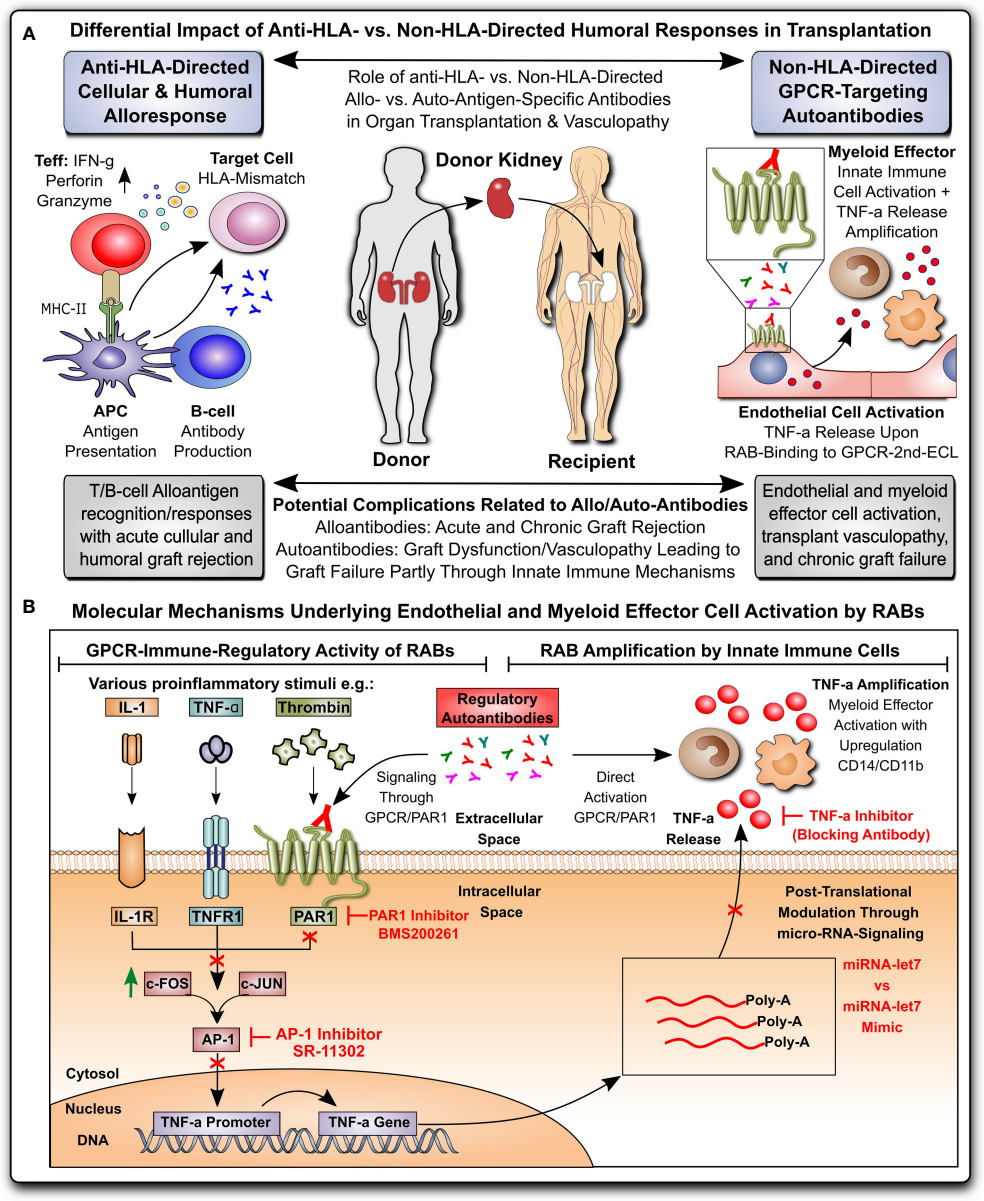
Non-HLA-directed autoantibodies from patients with kidney allograft vasculopathy stimulate a proinflammatory switch in endothelial cells and monocytes. **(A)** Differential impact of anti-HLA- vs. non-HLA-directed humoral responses in transplantation: Anti-HLA-directed cellular and humoral alloresponses are mainly effected by T- and B-cells and typically lead to acute humoral and cellular graft rejection in a mismatch transplant setting, while Non-HLA-directed G-protein coupled receptor (GPCR)-targeting regulatory autoantibodies (RAB) predominantly activate vasculature-resident GPCR-expressing endothelial cells to express TNF-α and subsequently activate innate immune effector, leading to chronic graft/transplant failure; and **(B)** Molecular mechanisms underlying endothelial and myeloid effector cell activation by RABs: ECs can be activated by various stimuli (e.g. TNF-α and other cytokines), or as shown here by GPCR-targeting RABs, which in the case of immunoglobulin G (IgG) isolated from transplant patients with transplant vasculopathy and rejection without detection of HLA-directed alloresponses (KTx-IgG), can bind to protease activated receptor 1 (PAR1; a GPCR) on the cell surface of ECs. Binding of KTx-IgG (containing the RABs), but not Con-IgG from healthy controls or KTx-IgG from patients without vasculopathy, activates the intracellular signaling cascades that lead to TNF-production, involving AP-1/c-FOS-signaling, TNF- promoter activation and further posttranscriptional regulation by microRNA-let7f-5g in ECs. The TNF-α secreted by KTx-IgG-activated ECs can kick of other secondary effects, such as THP-1 myeloid effector cell activation (detected by upregulation of surface receptors CD14 and CD11b on THP-1 cells), that may altogether contribute to the development of KTx transplant vasculopathy. AP-1, activator protein 1 transcription factor complex composed of subunits c-FOS and c-JUN; PAR-1, protease activated receptor 1; TNF-α, tumor necrosis factor alpha.

In the present study, we determined whether non-HLA autoantibodies isolated from the serum of KTx recipients with allograft vasculopathy can stimulate ECs and monocytes to release significant amounts of TNF-α. We found an RAB-dependent synergistic TNF-α release from ECs and monocytes mediated via the PAR-1 receptor on ECs (**Figure 1**).

## 2 Methods

### 2.1 KTx patient and healthy serum, IgG isolation, and materials

Blood serum samples from KTx patients or healthy controls were obtained upon written informed consent and ethical approval from the local review board at Charité in agreement with the declaration of Helsinki and respective IgG fractions isolated as detailed earlier (23, 37). The IgG’s were suspended in basic culture medium and stored at -80 degrees until use. The IgG for functional studies was prepared from n=7 patients with KTx-associated vasculopathy (KTx-IgG) and compared to IgG from n=7 healthy age and sex-matched controls (Con-IgG) and used in experiments at 1mg/ml as specified in the figure legends (**Table S1**). Unless stated otherwise, chemicals were from Sigma-Aldrich (Taufkirchen, Germany), and culture plastics were purchased from Corning and Becton Dickinson (Falcon; Franklin Lakes, NJ, USA). Cell culture media and buffers were from Thermo Fisher (Dreieich, Germany), and fetal calf serum (FCS) from Invitrogen (Darmstadt, Germany). The stimulators/inhibitors were as follows: thrombin (19), phorbol-myristate-acetate (PMA), activator protein 1 (AP1) inhibitor (SR-11302), and PAR1 inhibitor (BMS200261). The characteristics of any molecular detection or blocking antibodies employed in this article are given in the supplement (**Table S2**).

### 2.2 Culture of THP-1 and HMECs and flow cytometry analysis of CD14 and CD11b expression on THP-1 monocytic cells

The monocytic cell line THP-1 (Merck, Darmstadt, Germany) was cultured in RPMI1640-medium supplemented with 10% FCS and 1% Penicillin/Streptomycin. Human microvascular endothelial cells (HMECs; Catalogue no. CRL-3243) were purchased from ATCC® (Manassas, VA, USA) and used at passages 2-6 and cultured as described previously (19, 38–42).

**Primary effect:** To measure the direct stimulatory effect of KTx-IgG on HMECs and THP-1, the cells were stimulated for 1-24 hours with 1 mg/ml of KTx-IgG or Con-IgG with subsequent quantification of TNF-α mRNA and cytokine production.

**Secondary Effect:** The effect of IgG-stimulated TNF-α release from HMECs on THP-1 cells was assessed in three steps. First, to activate the HMECs, the cells were incubated for 6 hours either with or without KTx-IgG or Con-IgG (HMEC activation step) either in the presence or absence of anti-TNF-α blocking antibody or respective IgG isotype control (**Table S2**), as specified in more detail the figure legends. Second, this was followed by a brief washing step, to remove unbound IgG and thereby prevent autocrine effects of residual IgG on THP-1 cells. The stimulation medium was entirely removed, and the adherent cells briefly washed with IgG-free medium, to remove any remaining residual liquid that could contain unbound IgG. Third, “conditioning medium” was added to the washed cell layer of activated HMECs (anti-PAR1 autoantibody bound to PAR1 receptor on HMECs), to collect the HMEC secreted secretome (including HMEC-secreted TNF-α), to be used as follows.

**Coculture of THP-1 with HMEC-conditioned medium:** The KTx-IgG-stimulated and HMEC-conditioned medium was then added to the THP-1 cells at a ratio of 10% (v/v). After a 16-hour incubation, the THP-1 cells were assessed with flow cytometry for CD14 and CD11b surface expression, indicative of THP-1 and myeloid cell activation, as described earlier (CD11b more sensitive) (43). Upon the exposure of THP-1 cells to KTx-IgG or Con-IgG conditioned HMEC supernatants, the THP-1 cells were collected by careful mechanical agitation and resuspension, centrifuged for 5min at 1200 rpm, washed with PBS, and checked for viability by using the propidium iodide (PI) exclusion assay (43–45). For the antibody labeling, the THP-1 cells were placed on ice to prevent unspecific changes in CD14 and CD11b surface receptor expression and then labeled for 30 min in the dark with titrated antibodies (0.1 ug/ul) directed against CD14, CD11b, or respective isotype controls (all from Beckman Coulter GmbH, Krefeld Germany) (**Table S2**). The antibody-labeled THP-1 cells were then washed once more with PBS to remove unbound antibodies and fixed with 1% (v/v) of paraformaldehyde (PFA) diluted in PBS and subsequently analyzed with a flow cytometer (FACS Aria and FASC Calibur; Becton Dickinson, San Jose, CA, USA).

### 2.3 Detection of cellular TNF-α release with Quantikine ELISA

The concentration of TNF-α protein in the conditioned cell culture supernatants was measured with a high-sensitivity DuoSet immunoassay (Quantikine ELISA Kit; R&D Systems (Minneapolis, MN, USA). The detection limit was 0.049 to 1.6 pg/mL (40, 46).

### 2.4 Quantitative gene expression mRNA and micro-RNA analysis with qRT-PCR

The *TNF-α* gene and *ß2-microglobulin* (*β2M*) housekeeping gene expression was assessed with quantitative reverse transcription PCR (qRT-PCR) (38, 44, 46–48). Total RNA was extracted using the PerfectPure RNA kit (5 Prime, Hamburg, Germany), and RNA concentration and purity were estimated with a spectrophotometer (Nanodrop; ThermoFischerScientific). The obtained RNA was reverse transcribed into cDNA with random hexamer primers and qRT-PCR performed on a 7500 Fast Block real-time PCR system (Applied Biosystems). The specificity of the PCR reaction was verified with melting curve analysis and relative amount of transcript calculated with the cycle threshold method using the Applied Biosystems 7500 System v.1.2.3 software. Gene expression of the target gene was normalized to that of the housekeeping gene ß2M; Primers were as follows (**Table S3**): TNF-α (GeneBank NM_000594.3): forward (5’-GACAAGCCTGTAGCCCATGT-3’), reverse (5’-GAGGTACAGGCCCTCTGATG-3’); ß2M (GeneBank NM_004048.2): forward (5’-GTGCTCGCGCTACTCTCTCT-3’), reverse (5’-CGGCAGGCATACTCATCTTT-3’). For micro-RNA analysis, following cDNA synthesis using the miScript II RT Kit (Qiagen, Hilden, Germany), the expression of 84 miRNAs was analyzed with miSript™ miRNA PCR Array Kit (Qiagen) according to the manufacturer’s instructions. Six different snoRNA/snRNA (SNORD61, SNORD68, SNORD72, SNORD95, SNORD96A, and RNAU6-6P) were used as normalization controls. To mimic endogenous miRNA, 1 pmol of synthesized double-stranded has-let-7f-5p miScript miRNA mimic was transfected into cells using HiPerFect transfection reagent, as indicated per the manufacturer’s protocol. After 16 hours, the cells were treated with KTx-IgG for 24 hours, and TNF-α release was measured by ELISA, as indicated above.

### 2.5 DNA constructs, transient transfection, TNF promoter computational analysis, nuclear extracts, and electrophoretic mobility shift assay

The DNA constructs of pre-defined TNF-α promoter fragments (**Table S3**) were analyzed with Electrophoretic mobility shift assays (EMSA) as described in detail (46). The promoter fragments were first checked for correct segment length by restriction digest. The cells were seeded into 6-well culture plates for transient transfection studies, and transfections were performed at 70-80% cell confluence in the absence of serum using the TurboFect™ transfection reagent (Thermo Fisher). The HMECs were transfected with the TNF-α reporter or reference plasmids and assayed with the dual-luciferase reporter assay system (Promega) (49). The human *TNF-α* promoter region located at -2010 to +50 (GenBank NT_007592.15) was analyzed with PROMO virtual laboratory for presence/location of potential transcription factor binding sites: http://alggen.lsi.upc.es/cgi-bin/promo_v3/promo/promoinit.cgi?dirDB=TF_8.3.

Nuclear extracts were prepared using NE-PER Nuclear and Cytoplasmic Extraction Kit and oligonucleotide probes labeled with Biotin 3’ End DNA Labeling Kit (Thermo). For EMSA (38), the following probes were used (Regions of *TNF-α* promoter given in brackets, **Figure S3**): 5’-CCACACGGAGGCATCTGCACCCTC-3’ (-1275 to -1298). Each binding mixture (20 µl) contained 5 µg nuclear extract, 20 fmol labeled double-stranded probe, 1 µg poly-dI/dC, and 2 µl 10 x buffer all incubated at room temperature for 30 min. Protein-DNA complexes were analyzed by electrophoresis in 6% non-denaturing polyacrylamide gels and visualized with LightShift Chemiluminescent EMSA Kit (Thermo). The activity of NFkB and NFAT signaling was analyzed with EMSA and luciferase assays, respectively, as described previously (50, 51).

### 2.6 Statistics

Statistical analysis was performed using GraphPad Prism 6.05 software (GraphPad Software). The data were analyzed with the t-test or repeated measures analysis of variance. Results were expressed as means ± SD. Differences with a *p*-value <0.05 were considered significant.

## 3 Results

### 3.1 Graphical abstract and synopsis of the major study results

The study background and a summary of the molecular mechanisms identified in this study can be found in **Figure 1**. In brief, kidney transplant patient-derived IgG (KTx-IgG; from patients with allograft vasculopathy), but not KTx-IgG from patients without allograft vasculopathy or healthy-donor-derived control IgG (Con-IgG), stimulates GPCR-PAR-1-signaling-axis-dependent TNF-α secretion from HMECs and THP-1 monocytic cells. Notably, the KTx-IgG-stimulated TNF-α secretion from HMECs can polarize and amplify THP-1 monocyte activation in response to the HMEC secretome.

### 3.2 KTx-IgG increases TNF-α production by HMECs and THP-1 monocytic cells

**Figure 2:**
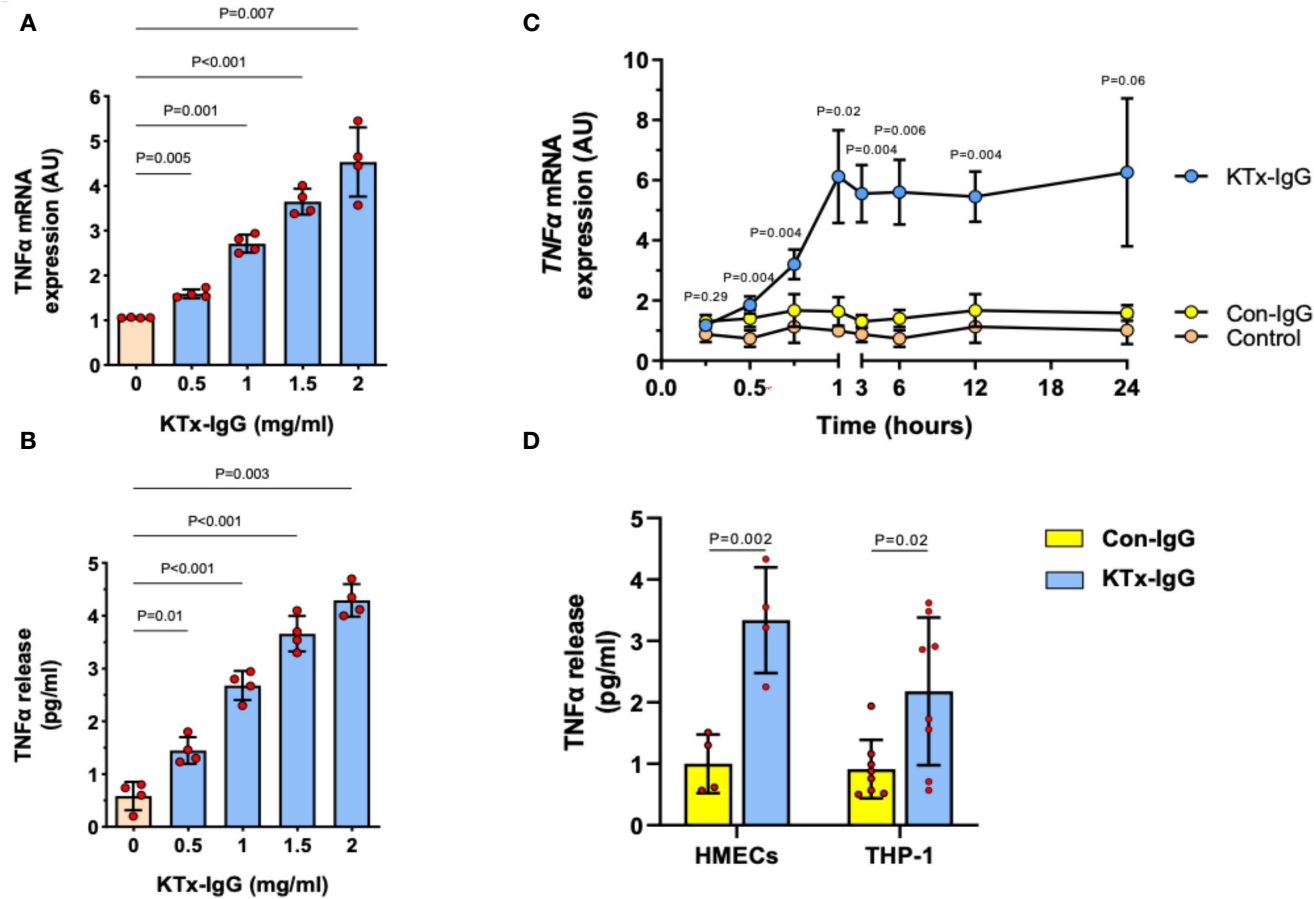
Effect of KTx-IgG on TNF-α production by HMECs. **(A, B)** Dose-response-effect of KTx-IgG on *TNF-α* mRNA expression and protein release from HMECs upon a 24-hour incubation with 0.5-2 mg/ml KTx-IgG (AU; arbitrary units, n=4); and **(C)** Kinetics of stimulation with KTx-IgG versus Con-IgG (each 1 mg/ml for 24 hours) on *TNF-α* mRNA expression in HMECs vs. unstimulated baseline (AU; depicting mean +/- SD of n=4); and **(D)** Direct comparison of KTx-IgG vs. Con-IgG (1 mg/ml for 24 hours) on TNF-α release (pg/ml) from both HMECs and THP-1 monocytes. The data were analyzed with repeated measures ANOVA [two-way ANOVA in **(C, D)**] with Šidáks multiple comparison test and are expressed as Mean +/- SD with *P<0.05, **P<0.01, and ***P<0.001.

We previously found that non-HLA autoantibodies target vasculature resident GPCRs to induce endothelial injury (1, 52). Here, we focused on the resulting inflammatory milieu. First, we found that the exposure of HMECs to the serum IgG fraction from patients with transplant vasculopathy (KTx-IgG) resulted in both a concentration- and time-dependent increase in *TNF-α* expression (P<0.05 to P<0.001; **Figures 2A–C**). This increase in *TNF-α* mRNA expression (P<0.01; **Figure 2A**) was accompanied by a similar KTx-IgG concentration-dependent increase in TNF-α protein release (P<0.01; **Figure 2B**). Importantly, KTx-IgG from patients without allograft vasculopathy did not stimulate a substantial increase in TNF-α protein release (P<0.05; **Figure S1A**). This increase in TNF-α occurred rapidly within the first 60 min of exposure and remained well above control levels for the next 12-24 hours, but this did not happen in HMECs treated with a control medium or with IgG from healthy control individuals (Con-IgG) (**Figure 2C**). To test whether the effect of KTx-IgG was cell-type-dependent, we studied the response of both HMECs and THP-1 monocytes (**Figure 2D**). Similar to HMECs, the exposure of THP-1 monocytes to KTx-IgG resulted in an increased TNF-α release compared to Con-IgG (P<0.01 vs. P<0.05; Mean increase in TNF-α from 1.0 pg/ml for Con-IgG to 3.5 and 2.2 pg/ml for KTx-IgG, respectively; **Figure 2D**).

### 3.3 KTx-IgG modulates TNF-α gene promoter activity in HMECs via AP1-/cFOS

**Figure 3:**
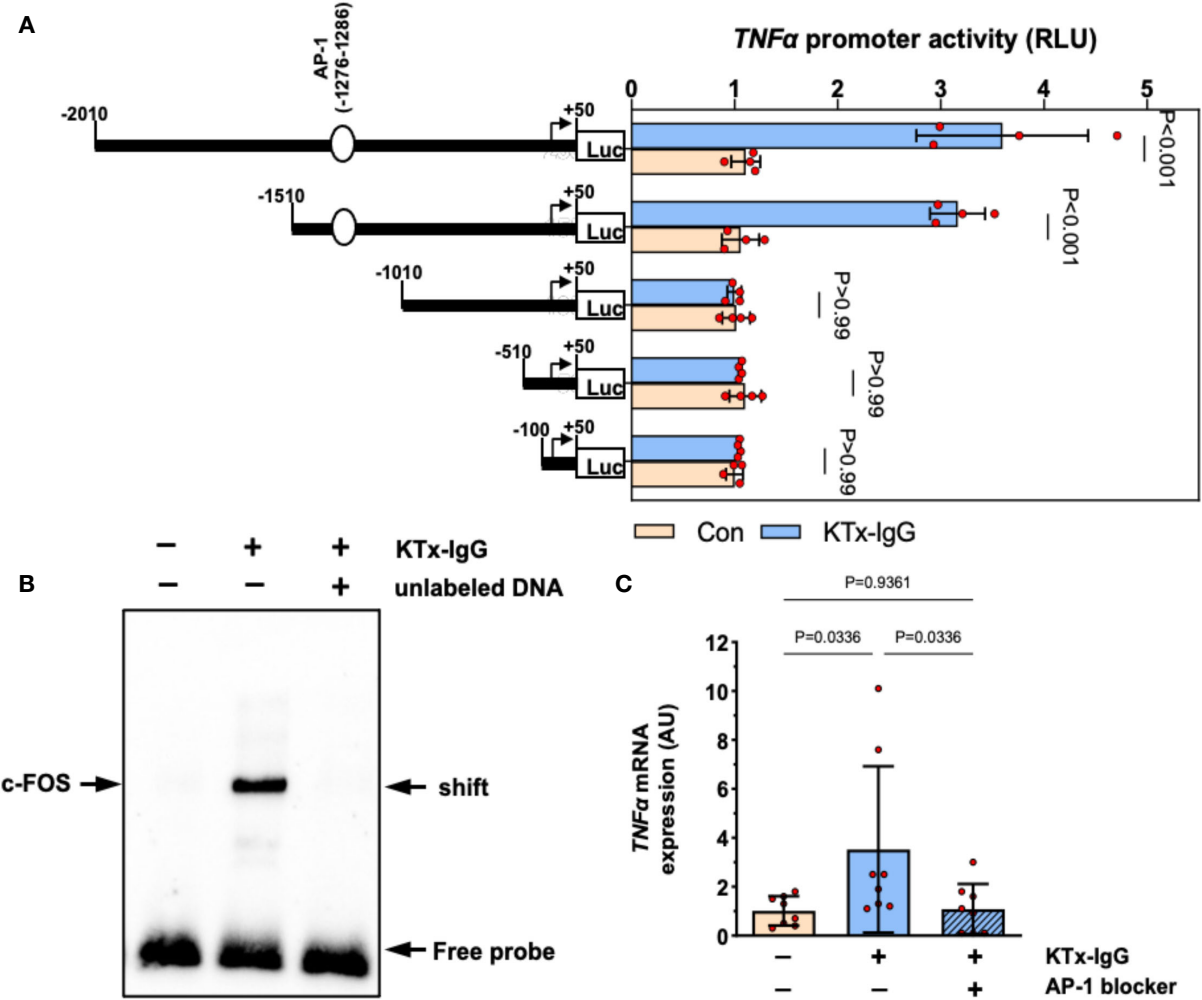
Transcriptional regulation of TNF-α expression by KTx-IgG in HMECs. **(A)** HMECs were transfected with either full-length TNF-α promoter construct or with its progressive 5’-deletions and then treated for 6 hours with or without KTx-IgG (1 mg/ml) and the activity of the luciferase reporter fragments was measured (RLI; relative luciferase activity; n=4, ANOVA), documenting that the TNF-α promoter activity is lost from the -1010-promoter-deletion onwards; **(B)** Nuclear fractions of HMECs stimulated as shown in **(A)** were analyzed by using EMSA with a biotin-labelled oligonucleotide with a predicted c-FOS binding sequence corresponding to the positions -1286 to -1277 of the TNF-α promoter. The EMSA was performed in the presence or absence of 100-fold molar excess of unlabeled oligonucleotide with one representative experiment is shown; and **(C)** TNF-α mRNA expression in HMECs stimulated for 12 hours with KTx-IgG (1 mg/ml) in the presence or absence of 1 µM AP-1-inhibitor SR-11302 (n=8; one-way ANOVA). The data are expressed as mean +/- SD with *P<0.05, **P<0.01, and ***P<0.001.

To elucidate the mechanism of KTx-IgG-induced modulation of *TNF-α* expression, HMECs were transfected with reporter constructs corresponding to different fragments of the *TNF-α* reporter, as shown earlier (19, 38, 40, 47, 53). First, we found that the activity of full-length *TNF-α* promoter construct (positions -2010 to +50) was substantially increased upon stimulation with KTx-IgG (P<0.001; **Figure 3A**) and that this activity was retained to position -1511 (P<0.001), while further truncation of the promoter to position -1011 abolished its activity in response to KTx-IgG (P>0.99). Subsequent *in silico* analysis pointed to the presence of high-affinity binding sites for the transcription factor c-FOS. To verify whether c-FOS mediated the effect of KTx-IgG towards the *TNF-α* promoter, an electrophoretic mobility shift assay (EMSA) was performed using a biotin-labeled consensus oligonucleotide for c-FOS binding that corresponded to positions -1286 to -1277 of the promoter (**Figure 3B**). This demonstrates that nuclear extracts from HMECs stimulated with KTx-IgG form a DNA-protein complex with the c-FOS-specific oligonucleotide. To verify the specificity of c-FOS binding, the EMSA was performed with a 100-fold molar excess of unlabeled oligonucleotide, which resulted in a loss of the c-FOS-DNA complex. The involvement of c-FOS in KTx-IgG-induced TNF-α mRNA expression was confirmed by blocking experiments with the AP-1/c-FOS-inhibitor SR-11302, leading to a strong reduction of TNF-α mRNA expression to baseline (P<0.05; **Figure 3C**). Additionally, we analyzed the potential activation of NFkB and NFAT signaling in HMECs in response to KTx-IgG from patients with vasculopathy with either EMSA or luciferase assays, respectively (**Figures S1C–D**). We did not find any substantial activation of either signaling pathway in response to KTx-IgG, e.g. no NFkB EMSA probe shift, but a clear EBNA positive control shift, and no increase in NFAT activity with KTx-IgG in comparison to Con-IgG.

### 3.4 KTx-IgG modulates the intrinsic expression of selected miRNAs in HMECs

**Figure 4:**
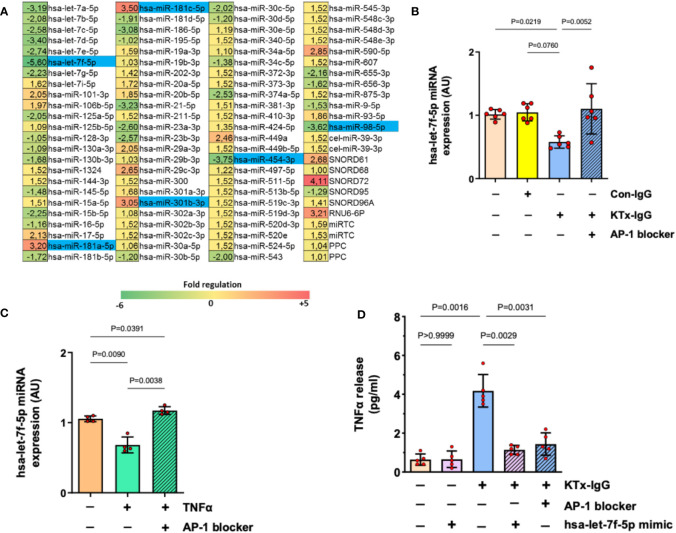
AP-1/miRNA-let-7f modulate KTx-IgG-induced TNF-production in HMECs. **(A)** HMECs were incubated for 1-hour with KTx-IgG (1 mg/ml) and miRNAs were isolated and analyzed using a profiler miRNA array, demonstrating no alterations, up- or down-regulation of specific targets (fold upregulation shown in red vs. downregulation shown in green), with the significantly changed targets marked in blue, identifying miRNA-hsa-let-7f-5p as the most strongly modulated target (-5.6 fold-downmodulation; for analysis of other targets please see **Figure S1**) while there were 2 more potential miRNA targets significantly downmodulated (-3.62 and -3.75) and three more upregulated (+3.05, +3.20, +3.50). **(B, C)** Expression analysis of miRNA-has-let-7f-5p in HMECs in the presence of AP-1 blocker (SR-11302, 1 µM). The cells were stimulated with either **(B)** KTx-IgG (1 mg/ml for 1 hour; n=6), or with **(C)** TNF-α (10 pg/ml for 1 hour; n=4), documenting a normalization/restoration of both KTx-IgG- and TNF-α-induced downmodulation in the presence of the AP-1 blocker. **(D)** Modulatory effect of miRNA-hsa-let-7f-5p and AP-1 on TNF-α production. HMECs were first transfected either with miRNA-hsa-let-7f-5p-mimic (1 pM) or pretreated with AP-1 blocker (SR-11302, 1 µM), and then stimulated with KTx-IgG (1 mg/ml) for 24 hours to quantify the TNF-α release from HMECs (pg/ml; n=5). Statistical testing was done with ANOVA and the data are depicted as mean +/- SD with *P<0.05, **P<0.01, and ***P<0.001.

To determine whether miRNAs mediate the effects of KTx-IgG, the expression of a well-defined panel of miRNAs was assessed in HMECs (**Figures 4**, **S2**).

Interestingly, along with the strong modulation of endothelial TNF-α expression, exposure to KTx-IgG (1 mg/ml) strongly modulated the expression of several miRNAs (**Figure 4A**), of whom six were selected for further analysis/validation (**Figure S2**). Remarkably, miRNA-hsa-let-7f-5p was consistently downregulated by KTx-IgG (P=0.08; **Figure 4B**), which was also documented upon stimulation with TNF-α (10 pg/ml) (P<0.01; **Figure 4C**). Interestingly, the downmodulation of miRNA-hsa-let-7f-5p by both KTx-IgG and TNF-α could be abolished by blocking AP-1 (both P<0.01; **Figures 4B, C**). To verify if the reduction in miRNA-hsa-let-7f-5p is linked to TNF-α production, the HMECs were transfected with miRNA-hsa-let-7f-5p-mimic (1 pM) and then stimulated with KTx-IgG (**Figure 4D**), which led to a substantial omission of KTx-IgG-mediated induction of TNF-α. Likewise, KTx-IgG did not increase TNF-α release in cells pretreated with AP-1 blocker.

### 3.5 KTx-IgG-induced TNF-expression in HMECs is mediated via PAR1

**Figure 5:**
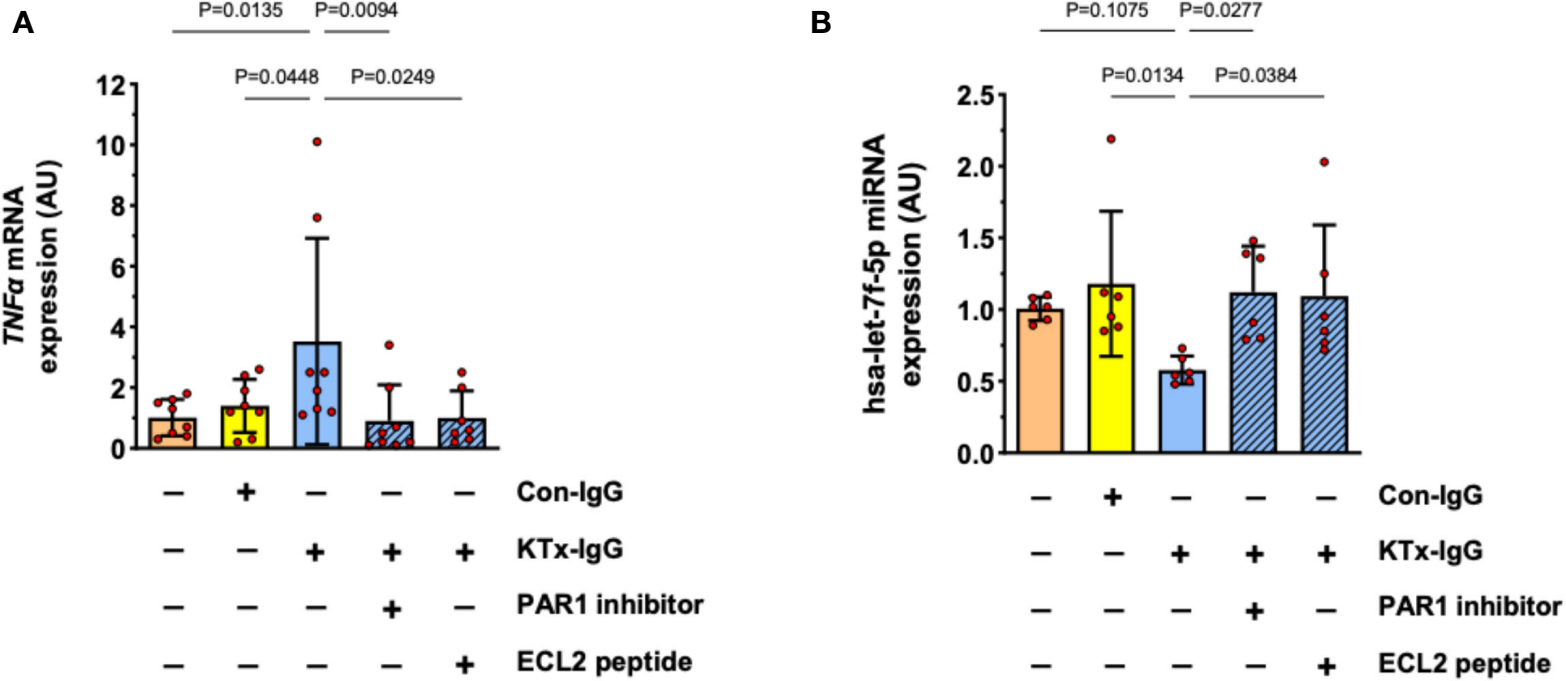
Role of cell surface receptor PAR-1 in KTx-IgG-induced responses in HMECs. The protease activated thrombin receptor PAR1 is a typical target for non-HLA autoantibodies contained in patient blood with disturbed autoantibody homeostasis (1, 19). To test whether PAR1 mediates the KTx-IgG-responses, the HMECs were stimulated for 3 hours with patient-derived KTx-IgG or health-donor-derived Con-IgG (both at 1 mg/ml) in the presence of either the PAR1 inhibitor (BMS200261, 1 µM), or small blocking peptide that is specific to the second extracellular loop of the PAR1 receptor (ECL2-specific peptide, 1 µM). After the exposure to the reagents, the HMECs were analyzed for: **(A)** TNF-α mRNA expression (AU, n=8), or **(B)** miRNA-hsa-let-7f-5p (AU, n=6), revealing a PAR1-dependent modulation of both targets. Statistical testing was done with ANOVA and the data are depicted as mean +/- SD with *P<0.05, **P<0.01, and ***P<0.001.

Previously, we have identified PAR1 as key mediator of HMEC responsiveness to extracellular stimuli (1, 19). Furthermore, PAR1 is an important target for non-HLA-directed autoantibodies contained in KTx-IgG that can bind to GPCRs (1, 19). To test if the effect of KTx-IgG is mediated via PAR1, HMECs were stimulated with KTx-IgG in the presence of BMS200261, a specific PAR1 blocker, or a competing peptide that corresponds to a sequence motif in the second extracellular loop (ECL2) of PAR1 (**Figure 5**). Indeed, both treatments abolished KTx-IgG-induced changes in both TNF-α mRNA (**Figure 5A**) and miRNA-hsa-let-7f-5p (**Figure 5B**), thus implying that the stimulatory effect of KTx-IgG on HMECs is mediated via the PAR1 GPCR surface receptor. Again, Con-IgG had no effect (**Figures 5A, B**), thereby iterating that the altered autoantibody homeostasis in KTx patients and their binding to GPCR receptors, such as PAR1 (in particular its ECL2) is crucial for the proinflammatory responses of HMECs. Importantly, the PAR1-mediated stimulatory effect of KTx-IgG was strongly amplified in the presence of its natural activator thrombin and PMA (2-3-fold increase, **Figure S1B**), and both were strongly reduced in the presence of PAR1-Inhibitor (both P<0.05; **Figure S1B**).

### 3.6 KTx-IgG-stimulated HMEC-derived TNF promotes THP-1 monocyte differentiation

**Figure 6:**
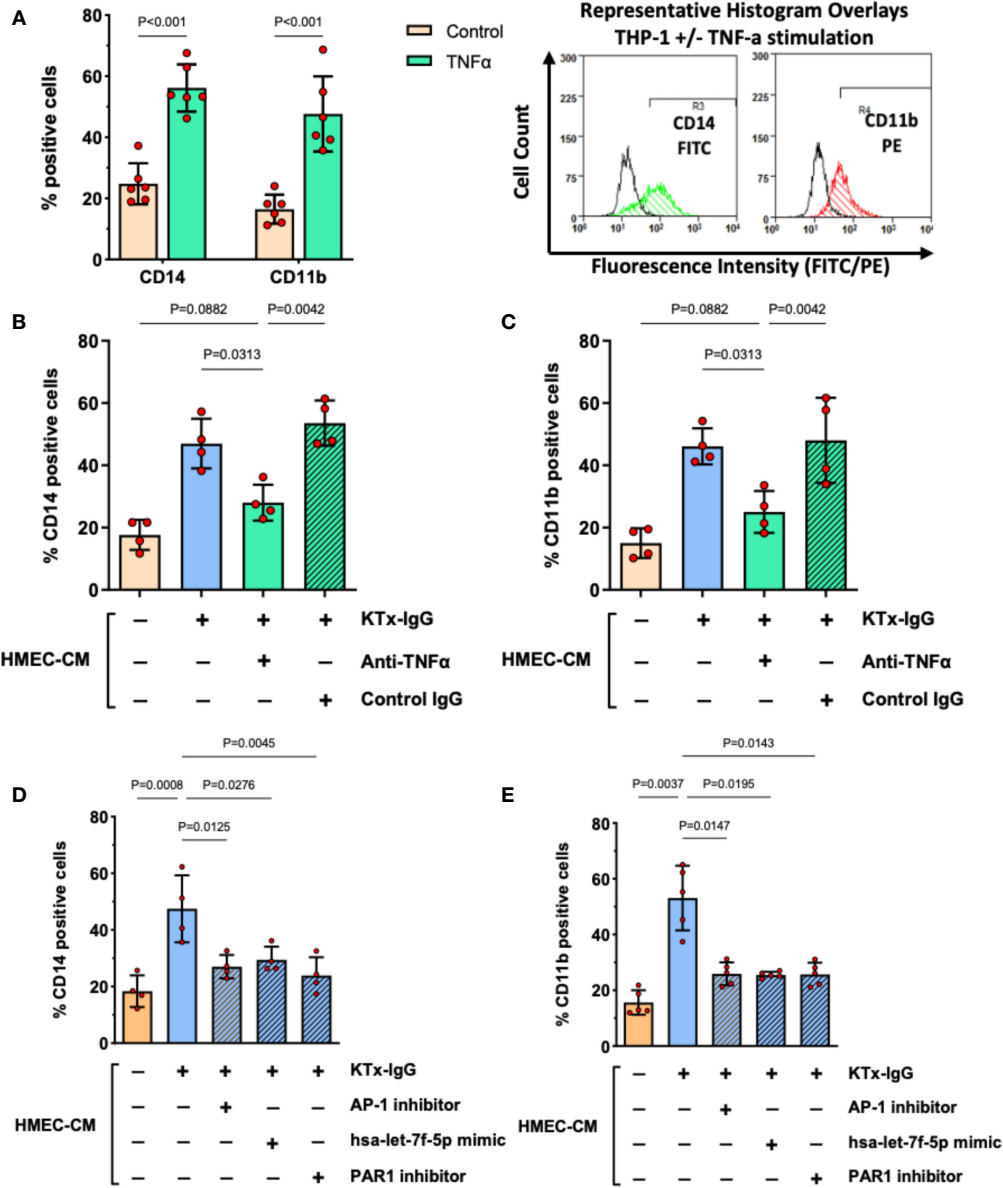
KTx-IgG-induced endothelial TNF-α stimulates THP-1 differentiation. **(A)** As positive control, THP-1 cells were treated for 16 hours with or without recombinant TNF-α (1 ng/ml) and then analyzed for the expression of monocyte activation markers CD14 and CD11b, indicating a doubling in the number of cells which are positive for these surface receptors (n=6) with representative histogram overlays shown to the right (CD14 and CD11b expression in TNF-α stimulated cells is depicted in green and red respectively, while the respective unstimulated cells are shown as black lines, in addition respective isotype controls were also included in the assay, not shown here). **(B, C)** The HMECs were preincubated with or without KTx-IgG (1 mg/ml) for 6 hours, washed to remove unbound KTx-IgG, and allowed to condition new culture medium for 24 hours with their secretome (HMEC-CM), which was then collected and added at a ratio of 10% of volume (v/v) together with TNF-α blocking or respective control antibody (Both at 1 µg/ml). After 16 hours of exposure, the THP-1 cells were assessed for either **(B)** CD14-FITC expression or **(C)** CD11b-PE expression with flow cytometry (n=4). **(D, E)** The HMECs were preincubated for 6 hours with or without KTx-IgG (1 mg/ml) in the presence or absence of AP-1 inhibitor (SR-11302, 1 µM), PAR1 inhibitor (BMS200261, 1 µM), or following miRNA-hsa-let-7f-5p-mimic transfection (1 pM). The cells were then washed and allowed to condition their culture media with their secretome for 24 hours (HMEC-CM), which was then collected and added to THP-1 cells at a ratio of 10% of volume (v/v) for 16 hours, after which the THP-1 cells were assessed for either **(D)** CD14-FITC, or **(E)** CD11b-PE expression with flow cytometry. The data in **(A)** were analyzed with t-test and in **(B–E)** with ANOVA, depicting mean +/- SD with *P<0.05, **P<0.01, and ***P<0.001.

Our previous work identified myeloid effector cell activation as an essential amplifier of mesenchymal and endothelial cell modulation of immune responses (34, 43). We employed a similar flow cytometry-based readout to study THP-1 responses to KTx-IgG-stimulated HMEC secretome (**Figure 6**). As a positive control, stimulation of THP-1 cells with recombinant TNF-α (1 ng/ml, 16 hours) resulted in a substantial increase/doubling in the expression of activated monocyte differentiation markers CD14 and CD11b (Both P<0.001; **Figure 6A**). To examine whether TNF-α released by KTx-IgG stimulated HMECs could elicit similar biological activity, THP-1 cells were exposed to HMEC conditioned medium, which also led to a similar increase in both CD14 and CD11b expression (Both P<0.05; **Figures 6B, C**). Importantly, this increase was abolished when the HMEC conditioned medium was treated with an anti-TNF-α antibody but not with the control antibody. Moreover, when the medium was conditioned by HMECs treated with KTx-IgG in the presence of AP-1 inhibitor, PAR1 blocker, or the miRNA-has-let-7f-5p-mimic, the expression of CD14 and CD11b on THP-1 cells was markedly reduced (All P<0.05; **Figures 2D, E**).

## 4 Discussion

The present study demonstrates that immunoglobulin G (IgG) antibodies derived from kidney transplant (KTx) recipients with allograft vasculopathy (KTx-IgG), but not IgG antibodies from healthy control individuals (Con-IgG) or KTx-IgG from patients without vasculopathy, are capable of stimulating TNF-α production in HMECs and monocytic cells (THP-1 model). This pathomechanisms may be involved in vascular inflammation during the development of allograft vasculopathy in the absence of donor-specific anti-HLA-antibodies (DSA) and cellular alloresponses (e.g., anti-HLA-directed effector T-cell responses). These anti-HLA directed alloresponses must be clearly distinguished from non-HLA-specific RABs previously identified in hallmark studies from our group to contribute to the pathomechanisms, which lead to non-HLA-related kidney allograft failure and allograft vasculopathy (1, 4, 5, 7–9, 11–15, 54, 55).

Noteworthy, this TNF-α stimulatory effect did only occur upon exposure of HMECs to KTx-IgG from transplant patients with underlying vasculopathy, which has been shown to exhibit disturbed homeostasis in their blood/vasculature-resident RABs (1), but not upon exposure to healthy control IgG (Con-IgG), or KTx-IgG from patients without vasculopathy. The RABs contained in KTx-IgG can not only promote G-protein-coupled receptor (GPCR) activation, but also lead to an altered expression of their highly potent GPCR effector targets (e.g. PAR1 upregulation) in the vasculature, which can then further augment/promote robust vascular responses upon RAB binding/engagement of their GPCR extracellular loop domains.

These detrimental events, the modulation of GPCR expression and its signaling intensity, can lead to kidney transplant rejection even in the absence of anti-HLA-directed alloantibodies (4). This pathomechanism may explain distinct cases of kidney allograft failure, where specific alloresponses have been ruled out as the underlying cause for allograft failure. Importantly, we here found here for the first time, that the nature of this TNF-α stimulatory effect is related to the ability of these RABs to induce signaling through the thrombin receptor PAR1, a typical protease activated GPCR (56–62).

One of the most important known natural activators of PAR1 is thrombin (19), which cleaves the n-terminus of the PAR1 receptor. Upon cleavage of the n-terminus, the tethered ligand can then bind to the PAR1 receptor to activate the intracellular signaling cascade. In contrast, the KTx-IgG activates the PAR1 receptor by binding to the second extracellular loop, which then leads to conformational changes of the 3D structure of the receptor in the cell membrane. Interestingly, we found that KTx-IgG from patients with vasculopathy increased the TNF-a response of HMECs to their natural activator thrombin and PMA.

Considering the biological *in vivo* relevance of the PAR1-KTx-IgG-signaling-axis-induced endothelial and monocytic TNF-α production and subsequent monocyte activation/differentiation in patients vs. *in vitro* coculture models, it needs to be anticipated that our observations are derived from a static *in vitro* coculture system, where both, the producer and the targets cells, are in close proximity to each other, which facilitates their interaction. It must also be noted that this coculture system is devoid of the vascular flow and fluid-transport condition (e.g. transport of blood or urine), as they are typically observed in the macro-micro-vasculature of the kidneys and kidney nephrons, respectively.

The binding of RABs to GPCRs and the subsequent induction of inflammatory responses may also be a common theme in pathologies other than kidney allograft failure, such as in patients undergoing transplantation of complex vascularized allografts (e.g., full hand Tx) (63), or in patients with dysregulated immune function (e.g., systemic sclerosis, SSc) (23). On the molecular level, RAB-binding to PAR1 resulted in an intracellular signaling cascade involving the transcription factor AP-1 and modulation of miRNA-hsa-let-7f-5p. Indeed, we have previously shown that the AP-1/c-FOS transcription factor complex is a prime relay/modulator of EC inflammatory responses in the vasculature (19, 40, 47).

The dysregulated activity of PARs has been implicated in many different diseases and pathological settings, as reviewed in detail in the following articles (56–62). In addition to serving as a prototypic receptor for its main activating ligand thrombin (19), PAR1 can also respond to other proteases, including plasmin, activate protein C, and matrix-metalloproteinases, to produce either pro-inflammatory or cytoprotective downstream effects, depending on the exact pathophysiological context, as discussed earlier (19). In this respect, we have previously reported an example of biased agonism exerted by IgG from patients with scleroderma (SSc-IgG), which was found to induce IL-6 expression in ECs by signaling through PAR1 (23).

Another exciting aspect of our current study is that we identified HMECs as a source of biologically active TNF-α in the vascular system in the context of binding and stimulation through EC surface resident GPCRs that bind RABs contained in the KTx-IgG but not in Con-IgG from healthy controls. Importantly, TNF-α activation/secretion of ECs will only become evident in the physiological or better said pathophysiological setting *in vivo* when the RAB network homeostasis is somehow disturbed in a particular disease or pathological setting (1). Again, the above-mentioned study on SSc-IgG is exemplary to illustrate how binding of RABs to GPCRs, such as PAR1, can trigger secretion of proinflammatory cytokines, such as IL-6, or in the current KTx-IgG setting secretion of TNF-α from HMECs.

The observation that HMECs can secrete TNF-α upon binding of KTx-IgG to their cell surface resident GPCRs is of some biological importance since ECs form the inner lining of the vasculature, measuring 300 to 1000 m2 at the blood-endothelial interface (64, 65). This may imply that the large endothelial surface can respond with TNF-α production in the vasculature, even though the amount secreted per cell is lower than that of immune cells typically implied as source of TNF-α, such as activated macrophages and T-cells (66). This is important, since ECs are not commonly associated with TNF-α secretion, although they have been shown to produce TNF-α upon pathological stimulation (35, 36).

Thus, our finding that ECs can produce TNF-α upon binding of RABs to their GPCRs adds to earlier reports on TNF-α production by ECs exposed to other proinflammatory cytokines or combinations thereof (35, 36). While the quantities of TNF-α released by KTx-IgG-activated ECs seem to be in the low picogram range, these levels may still be of biological significance. Indeed, we could show here that KTx-IgG-activated HMEC-conditioned medium contained TNF-α at such concentrations, that it can promote activation/differentiation of myeloid cells to activated monocytes/macrophages, but that this effect could be abolished by neutralizing TNF-α in HMEC-conditioned culture media.

Moreover, we were able to delineate some of the underlying signaling pathways leading to TNF-α induction in HMECs and, subsequently monocytes in response to the TNF-α secreted by the HMECs. These signaling events involved the transcription factor AP-1 and miRNA-hsa-let-7f-5p. We have previously demonstrated that the induction of IL-6 by antibodies that target PAR1 in ECs is also controlled by AP-1 (23). This indicates that the early response element AP-1 may act as an early master regulator in the events elicited by KTx-IgG-derived anti-PAR1-RABs. The here-identified involvement of miRNA-hsa-let-7f-5p in underlying anti-PAR1-induced RAB-signaling cascades has not been reported previously and is thus of interest as a mechanistic target for further studies.

Indeed, miRNAs and other noncoding RNAs have been identified as essential modulators of signaling processes in the vasculature, particularly in the context of tissue ischemia/hypoxia and related angiogenic responses (67, 68). MicroRNAs are single-stranded, small noncoding RNAs that can act at the post-transcriptional level to activate or suppress the expression of specific target genes (69). We here found that the stimulation of HMECs with KTx-IgG resulted in the modulation of several miRNAs, but in particular, in a substantial decrease in miRNA-hsa-let-7f-5p, which appeared to be the best target in our subsequent mechanistic validation.

In contrast, supplementation of sufficient amounts of synthetic “miRNA-mimic” agent to replenish the cell endogenous miRNA-hsa-let-7f-5p abolished the stimulating effect of KTx-IgG on TNF-α production. Accordingly, the conditioned medium from HMECs treated with KTx-IgG in the presence of miRNA-hsa-let-7f-5p-mimic did not induce TNF-α-dependent effects on monocytes. Interestingly, the downmodulatory effect of KTx-IgG on miRNA-hsa-let-7f-5p expression could be enhanced by the addition of TNF-α itself, thereby suggesting a feedforward interaction.

Although known for over two decades, let-7 miRNA remains a puzzle (70). In the past, it has been predominantly linked to the development of various cancers but also with the regulation of innate immune responses (70). Our new observations may support the involvement of let-7 miRNA in inflammation and innate immune responses, in which TNF-α and particularly activated monocytes/macrophages are key participants. In this regard, it has been demonstrated earlier that depleting/reducing the number of monocytes and macrophages in animal models can minimize microvascular dysfunction in transplanted kidneys (71, 72).

One of the most promising implications of our findings concerns early disease detection. The correlation between RAB-levels and TNF-α in (kidney) allograft vasculopathy patients should be confirmed in larger clinical studies and potentially serve as valuable, non-invasive biomarkers for monitoring graft health and failure risk (23, 73–75).

The identification of the PAR1-AP-1/c-FOS-miRNA-let7-axis in our study points out the possibility for targeted therapies to minimize the risk of transplant vasculopathy. For instance, Vorapaxar, an FDA-approved PAR1 inhibitor used in cardiovascular events, could be repurposed for transplant patients (76, 77). Similarly, AP-1/c-FOS inhibitors like T-5224 showing promising results and could be explored in this context (78). Furthermore, gene therapies focusing on miRNA let-7 modulators, which are mostly in the research phase for various diseases, could be a novel therapeutic approach (79, 80). On the intercellular level, existing TNF-α inhibitors like the well-studied Infliximab, approved for conditions such as rheumatoid arthritis, offer a promising therapeutic intervention (81, 82). Therefore, our study adds new possibilities for therapeutic interventions and potential targeted therapies in allograft vasculopathy.

## 5 Conclusions

Our here presented findings suggest that non-HLA-directed GPCR/PAR1-activating regulatory autoantibodies (RABs) that are found in KTx-IgG from transplant patients with vasculopathy can trigger substantial TNF-α production from ECs and myeloid cells, and KTx-IgG from patients with vasculopathy potentiates the response to the natural activator thrombin. Apart from the myeloid TNF-α production, the EC-derived TNF-α production can further promote monocyte activation and differentiation. The here identified detrimental amplification loop induced by GPCR-binding RABs may contribute to allograft vasculopathy, and thus graft failure/rejection irrespective of alloimmuneresponses.

Considering the novel observation of TNF-α production by HMECs; First, this appears to be specific to PAR1-directed stimulation of HMECs with KTx-IgG from patients with underlying kidney graft vasculopathy, which is not a common experimental setting, and may thus not have been noted in earlier studies, and thus this appears to be a very novel finding. For assurance we have also provided the concomitant intracellular signaling analysis. Second, the quantity of TNF-α released by HMEC cells upon stimulation with KTx-IgG is very low (depending on the experimental setting less than 5 pg/mL) and thus it is difficult to detect in the first place, but nonetheless this amount may still be significant. While these levels are very low, they may still be of biological significance as: i) the endothelial surface in humans is very large, and ii) KTx-IgG-activated HMEC-conditioned medium promoted activation/differentiation of myeloid cells *in vitro* and that this effect can be abolished by neutralizing TNF-α.

In contrast to the well-recognized aspects of adaptive immunity in transplantation, the role of innate immunity and humoral autoimmune responses has been somewhat neglected in the past since the activation of its components has been classically seen rather as a consequence than a cause of (subsequent) lymphocyte activation. In line with a few earlier reports (83–85), the here presented new results indicate an often-underestimated role of the innate immune system in transplant rejection that may deserve more attention and further studies. Our new results may lead to a better mechanistic understanding of kidney transplant vasculopathy and thereby reduce any associated detrimental outcomes for patients. Although the relevant HLA barriers and their associated alloimmuneresponses are of predominant importance in the kidney allograft transplantation setting, we here urge our readers to also consider the potential role of the innate immune responses as a potential negative confounder in the transplant setting, that should not be forgotten.

Further research in this direction may yield fruitful new insights into the regulation of vascular immune homeostasis, particularly on the role of vasculature resident RABs and their targeted GPCRs, to better understand and improve clinical outcomes (1).

## Data availability statement

The original contributions presented in the study are included in the article/**Supplementary Material**. Further inquiries can be directed to the corresponding authors.

## Ethics statement

The studies involving humans were approved by the local review board at Charité Universitätsmedizin Berlin. The studies were conducted in accordance with the local legislation and institutional requirements. The participants provided their written informed consent to participate in this study.

## Author contributions

GM: Conceptualization, Data curation, Formal Analysis, Funding acquisition, Investigation, Methodology, Resources, Software, Supervision, Visualization, Writing – original draft, Writing – review & editing. CL: Conceptualization, Data curation, Formal Analysis, Investigation, Methodology, Software, Validation, Visualization, Writing – original draft, Writing – review & editing. MG: Conceptualization, Data curation, Formal Analysis, Investigation, Methodology, Software, Writing – original draft, Writing – review & editing, Validation, Visualization. DF: Data curation, Formal Analysis, Investigation, Methodology, Software, Visualization, Writing – original draft, Writing – review & editing, Conceptualization, Validation. PW: Data curation, Formal Analysis, Investigation, Methodology, Conceptualization, Writing – review & editing. HZ: Data curation, Formal Analysis, Investigation, Methodology, Conceptualization, Writing – review & editing. ZG: Data curation, Formal Analysis, Investigation, Methodology, Conceptualization, Writing – review & editing. LC: Data curation, Formal Analysis, Investigation, Methodology, Conceptualization, Writing – review & editing. MA: Conceptualization, Formal Analysis, Funding acquisition, Investigation, Methodology, Resources, Data curation, Writing – review & editing. HH: Data curation, Formal Analysis, Funding acquisition, Investigation, Methodology, Resources, Conceptualization, Writing – review & editing. AH: Conceptualization, Data curation, Formal Analysis, Funding acquisition, Investigation, Methodology, Resources, Writing – review & editing. DD: Conceptualization, Funding acquisition, Investigation, Resources, Supervision, Data curation, Formal Analysis, Methodology, Writing – review & editing. KB: Conceptualization, Funding acquisition, Investigation, Resources, Supervision, Data curation, Formal Analysis, Methodology, Writing – review & editing. OP: Conceptualization, Funding acquisition, Investigation, Methodology, Resources, Supervision, Writing – review & editing, Data curation, Formal Analysis. GR: Conceptualization, Funding acquisition, Investigation, Methodology, Resources, Supervision, Data curation, Formal Analysis, Writing – review & editing. OC-M: Conceptualization, Data curation, Formal Analysis, Funding acquisition, Investigation, Methodology, Resources, Supervision, Visualization, Writing – original draft, Writing – review & editing, Software, Validation, Project administration. JW: Conceptualization, Data curation, Formal Analysis, Funding acquisition, Investigation, Methodology, Project administration, Resources, Software, Supervision, Validation, Visualization, Writing – original draft, Writing - review & editing. RC: Conceptualization, Data curation, Formal Analysis, Funding acquisition, Investigation, Methodology, Project administration, Resources, Software, Supervision, Validation, Visualization, Writing – original draft, Writing – review & editing.

## Supplementary material

Supplementary Table 1 KTx patient and healthy donor serum and IgG samples.

Supplementary Table 2 Antibodies and reagents used for flow cytometry and blocking antibody.

Supplementary Table 3 Sequences of primers used in quantitative-real-time-PCR analysis.

Supplementary Figure 1 Effect of KTx-IgG on HMEC TNF-α production and NFkB/NFAT activation. **(A, B)** Effect of KTx-IgG on TNF-α protein release from HMECs **(A)** Upon a 24-hour incubation with 1.0 mg/ml Con-IgG or KTx-IgG from patients with or without vasculopathy or baseline control (pg/ml, n=3 each) and **(B)** Upon a 24-hour stimulation with 1 mg/ml KTx-IgG from patients with vasculopathy (n=5 each) in the presence or absence of the following stimuli: a) mitogen phorbol-myristate-acetate (PMA, concentration 50 ng/ml), b) thrombin (0.1 U/ml), and c) PAR-1 inhibitor (BMS200261, 1 µM); and **(C, D)** Analysis of NFkB and NFAT activation in response to KTx-IgG from patients with vasculopathy compared to Con-IgG (each 1 mg/ml for 24 hours). The activity of NFkB and NFAT signaling was analyzed with EMSA and luciferase assays, respectively, as described previously (50, 51). The data were analyzed with repeated ANOVA and expressed as Mean +/- SD with *P<0.05, **P<0.01, and ***P<0.001.

Supplementary Figure 2 Supplement to **Figure 4**.

Supplementary Figure 3 Supplement to **Figure 3** .The TNF-α promoter sequence with the c-FOS binding sites marked in Red, respectively: -1509 ATTTATGAAGGCAAAAAAATTAAATTATTTATTTATGGAGGATGGAGAGAGGGGAATAATAGAAGAACATCCAAGGAGAAACAGAGACAGGCCCAAGAGATGAAGAGTGAGAGGGCATGCGCACAAGGCTGACCAAGAGAGAAAGAAGTAGGCATGAGGGATCACAGGGCCCCAGAAGGCAGGGAAAGGCTCTGAAAGCCAGCTGCCGACAGAGCCCCACACGGAGGCATCTGCACCCTCGATGAAGCCCAATAAACCTCTTTTCTCTGAAATGCTGTCTGCTTGTGTGTGTGTGTCTGGGAGTGAGAACTTCCCAGTCTATCTAAGGAATGGAGGGAGGGACAGAGGGCTCAAAGGGAGCAAGAGCTGTGGGGAGAACAAAAGGATAAGGGCTCAGAGAGCTTCAGGGATATGTGATGGACTCACCAGGTGAGGCCGCCAGACTGCTGCAGGGGAAGCAAAGGAGAAGCTGAGAAGATGAAGGAAAAGTCAGGGTCTGGAGGGGCGGGGGTCAGGGAGCTCCTGGGAGATATGGCCACATGTAGCGGCTCTGAGGAATGGGTTACAGGAGACCTCTGGGGAGATGTGACCACAGCAATGGGTAGGAGAATGTCCAGGGCTATGGAAGTCGAGTATGGGGACCCCCCCTTAACGAAGACAGGGCCATGTAGAGGGCCCCAGGGAGTGAAAGAGCCTCCAGGACCTCCAGGTATGGAATACAGGGGACGTTTAAGAAGATATGGCCACACACTGGGGCCCTGAGAAGTGAGAGCTTCATGAAAAAAATCAGGGACCCCAGAGTTCCTTGGAAGCCAAGACTGAAACCAGCATTATGAGTCTCCGGGTCAGAATGAAAGAAGAAGGCCTGCCCCAGTGGGGTCTGTGAATTCCCGGGGGTGATTTCACTCCCCGGGGCTGTCCCAGGCTTGTCCCTGCTACCCCCACCCAGCCTTTCCTGAGGCCTCAAGCCTGCCACCAAGCCCCCAGCTCCTTCTCCCCGCAGGGACCCAAACACAGGCCTCAGGACTCAACACAGCTTTTCCCTCCAACCCCGTTTTCTCTCCCTCAAGGACTCAGCTTTCTGAAGCCCCTCCCAGTTCTAGTTCTATCTTTTTCCTGCATCCTGTCTGGAAGTTAGAAGGAAACAGACCACAGACCTGGTCCCCAAAAGAAATGGAGGCAATAGGTTTTGAGGGGCATGGGGACGGGGTTCAGCCTCCAGGGTCCTACACACAAATCAGTCAGTGGCCCAGAAGACCCCCCTCGGAATCGGAGCAGGGAGGATGGGGAGTGTGAGGGGTATCCTTGATGCTTGTGTGTCCCCAACTTTCCAAATCCCCGCCCCCGCGATGGAGAAGAAACCGAGACAGAAGGTGCAGGGCCCACTACCGCTTCCTCCAGATGAGCTCATGGGTTTCTCCACCAAGGAAGTTTTCCGCTGGTTGAATGATTCTTTCCCCGCCCTCCTCTCGCCCCAGGGACATATAAAGGCAGTT GTTGGCACACCCAgccagcagacgctccctcagcaaggacagcagaggaccagctaagaggga +50. **Legend:**ATTTATGAAGGCA…TNF-α promoter sequence (-1509bp shown) GAGGCATCTGcFOS binding site (-1277bp bis -1286bp) TaagagggaTNFa gene sequence.
