# Supplementary material for: Autoantibodies from patients with kidney allograft vasculopathy stimulate a proinflammatory switch in endothelial cells and monocytes mediated via GPCR-directed PAR1-TNF-α signaling
Source: Front Immunol. 2023 Oct 30;14:1289744. doi: 10.3389/fimmu.2023.1289744 (PMC10642342; doi:10.3389/fimmu.2023.1289744)
Supplement: Supplementary Table 1 — KTx patient and healthy donor serum and IgG samples. [file Table_1.docx]

**Table S1: KTx patient and healthy donor serum and IgG samples**

|  | **KTx-IgG** | **Con-IgG** |
| --- | --- | --- |
| **Donor Sex (M/F)** | 5/2 | 3/4 |
| **Median Donor Age (Y, Range)** | 53 (37-69) | 40 (31-53) |
| **Patient Description** | KTx with Vasculopathy Anti-HLA-Negative | Healthy donors |

**Table S2: Antibodies and reagents used for flow cytometry and blocking antibody.**

| **FACS Antibody** | **Target Antigen** | **Host** | **Code** | **Company** | **Dilution** |
| --- | --- | --- | --- | --- | --- |
| **CD14**  **(PE-Conj.)** | Anti-human CD14  (IgG-2a Mouse) | Mouse  Monoclonal | B36297 | Beckman  Coulter | 1:500 |
| **CD11b**  **(FITC-Conj.)** | Anti-human MAC1a  (IgG1 Mouse) | Mouse  Monoclonal | IM0530 | Beckman  Coulter | 1:500 |
| **Isotypes**  **(FITC/PE-Con)** | Mouse IgG1-FITC /  IgG2a Isotype-PE | Mouse  Monoclonal | A07795 | Beckman  Coulter | 1:500 |
| **TNF-alpha Neutralizing Antibody** | Anti-human TNFα | Monoclonal Rabbit IgG Clone #R101 | 10602-HNAE | Sino Biological | 0.25ng/ml |

**Table S3: Sequences of primers used in quantitative-real-time-PCR analysis.**

| **Gene** | **Sequence Sense Primer 5’ to 3’** | **Sequence Antisense Primer 3’ to 5’** |
| --- | --- | --- |
| **B2M** | GTGCTCGCGCTACTCTCTCT | CGGCAGGCATACTCATCTTT |
| **TNF-α** | gACAAgCCTgTAgCCCATgT | gAggTACAggCCCTCTgATg |
| **c-FOS** | AGGAGAATCCGAAGGGAAAG | CTTCTCCTTCAGCAGGTTGG |
| **AP-1**  **Oligo** | CCACACGGAGGCATCTGCACCCTC | GAGGGTGCAGATGCCTCCGTGTGG |
| *pLuc*  *2060* | TGGCCTAACTGGCCGGTACCGCAGGAACCCTGGCTGCA | TCTTGATATCCTCGAGTCCCTCTTAGCTGGTCCT |
| *pLuc*  *1560* | TGGCCTAACTGGCCGGTACCATTTATGAAGGCAAAAAAATTAAA | Same sequence as pLuc 2060 above |
| *pLuc*  *1060* | TGGCCTAACTGGCCGGTACCGAGGGGCGGGGGTCAGGG | Same sequence as pLuc 2060 above |
| *pLuc*  *560* | TGGCCTAACTGGCCGGTACCGCAGGGACCCAAACACAG | Same sequence as pLuc 2060 above |
| *pLuc*  *150* | TGGCCTAACTGGCCGGTACCATTCTTTCCCCGCCCTCC | Same sequence as pLuc 2060 above |

**Abbreviations S3:** B2M, beta-2 microglobulin; TNF-α, tumor-necrosis factor 1-alpha; AP-1, activator protein-1; c-FOS, proto-oncogene subunit of the AP-1 transcription factor complex; and Oligo, oligonucleotide.
