# Supplementary figures and images for: Autoantibodies from patients with kidney allograft vasculopathy stimulate a proinflammatory switch in endothelial cells and monocytes mediated via GPCR-directed PAR1-TNF-α signaling
Source: Front Immunol. 2023 Oct 30;14:1289744. doi: 10.3389/fimmu.2023.1289744 (PMC10642342; doi:10.3389/fimmu.2023.1289744)

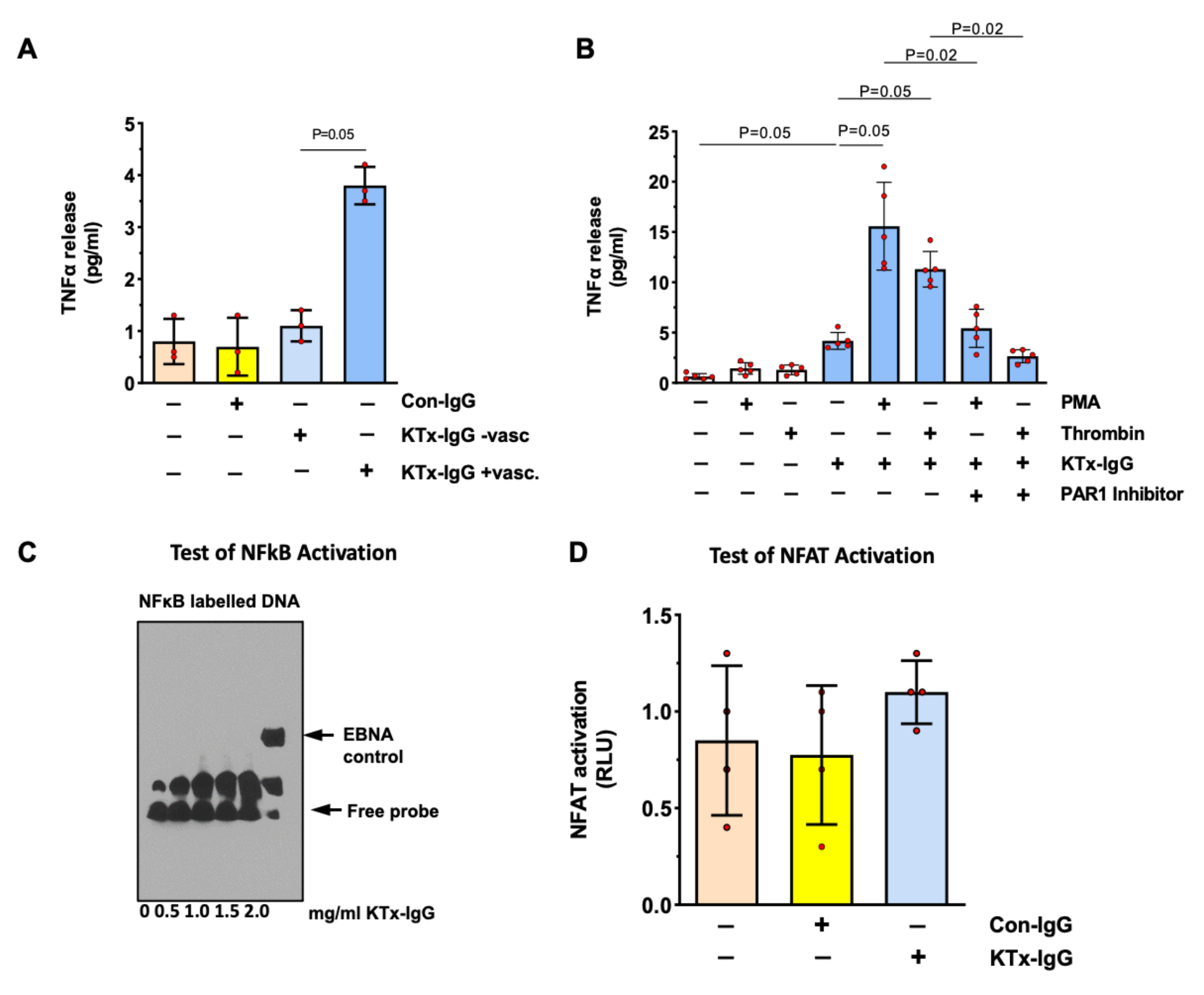

Supplement: Supplementary Figure 1 — Effect of KTx-IgG on HMEC TNF-α production and NFkB/NFAT activation. (A, B) Effect of KTx-IgG on TNF-α protein release from HMECs (A) Upon a 24-hour incubation with 1.0 mg/ml Con-IgG or KTx-IgG from patients with or without vasculopathy or baseline control (pg/ml, n=3 each) and (B) Upon a 24-hour stimulation with 1 mg/ml KTx-IgG from patients with vasculopathy (n=5 each) in the presence or absence of the following stimuli: a) mitogen phorbol-myristate-acetate (PMA, concentration 50 ng/ml), b) thrombin (0.1 U/ml), and c) PAR-1 inhibitor (BMS200261, 1 µM); and (C, D) Analysis of NFkB and NFAT activation in response to KTx-IgG from patients with vasculopathy compared to Con-IgG (each 1 mg/ml for 24 hours). The activity of NFkB and NFAT signaling was analyzed with EMSA and luciferase assays, respectively, as described previously (50, 51). The data were analyzed with repeated ANOVA and expressed as Mean +/- SD with *P<0.05, **P<0.01, and ***P<0.001. [file Image_1.tif]

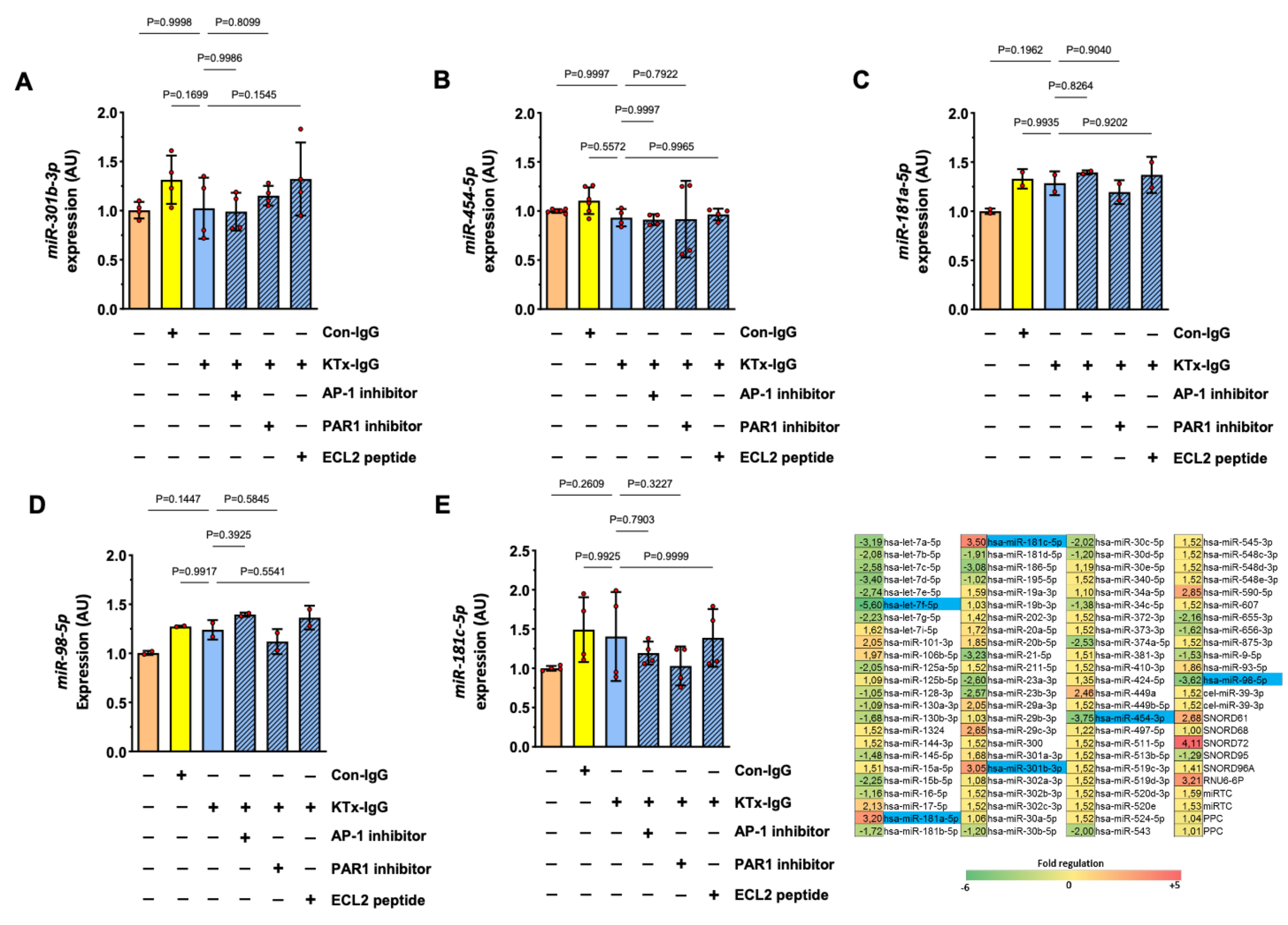

Supplement: Supplementary Figure 2 — Supplement to Figure 4 . [file Image_2.tif]

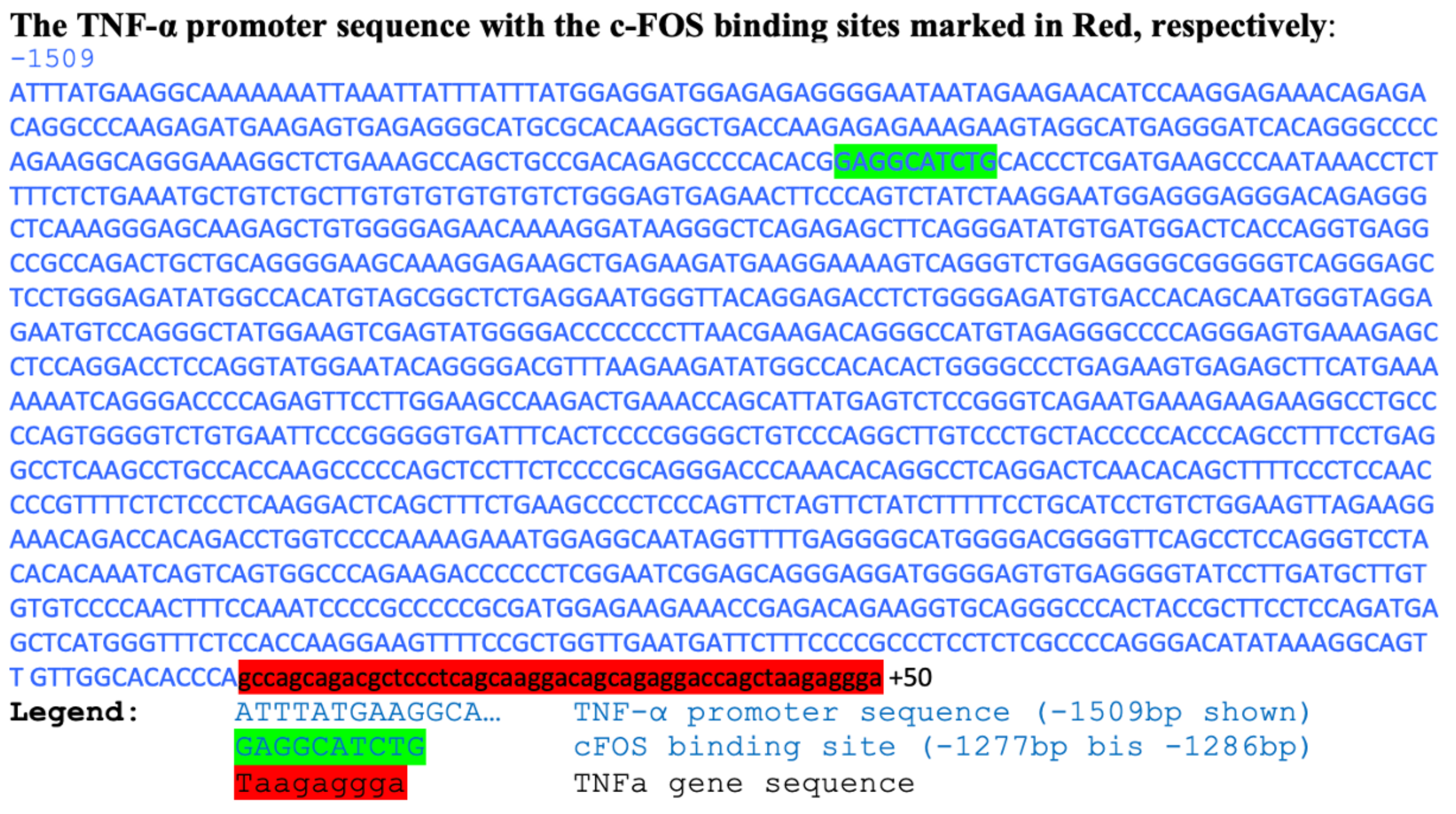

Supplement: Supplementary Figure 3 — Supplement to Figure 3 .The TNF-α promoter sequence with the c-FOS binding sites marked in Red, respectively: -1509 ATTTATGAAGGCAAAAAAATTAAATTATTTATTTATGGAGGATGGAGAGAGGGGAATAATAGAAGAACATCCAAGGAGAAACAGAGACAGGCCCAAGAGATGAAGAGTGAGAGGGCATGCGCACAAGGCTGACCAAGAGAGAAAGAAGTAGGCATGAGGGATCACAGGGCCCCAGAAGGCAGGGAAAGGCTCTGAAAGCCAGCTGCCGACAGAGCCCCACACGGAGGCATCTGCACCCTCGATGAAGCCCAATAAACCTCTTTTCTCTGAAATGCTGTCTGCTTGTGTGTGTGTGTCTGGGAGTGAGAACTTCCCAGTCTATCTAAGGAATGGAGGGAGGGACAGAGGGCTCAAAGGGAGCAAGAGCTGTGGGGAGAACAAAAGGATAAGGGCTCAGAGAGCTTCAGGGATATGTGATGGACTCACCAGGTGAGGCCGCCAGACTGCTGCAGGGGAAGCAAAGGAGAAGCTGAGAAGATGAAGGAAAAGTCAGGGTCTGGAGGGGCGGGGGTCAGGGAGCTCCTGGGAGATATGGCCACATGTAGCGGCTCTGAGGAATGGGTTACAGGAGACCTCTGGGGAGATGTGACCACAGCAATGGGTAGGAGAATGTCCAGGGCTATGGAAGTCGAGTATGGGGACCCCCCCTTAACGAAGACAGGGCCATGTAGAGGGCCCCAGGGAGTGAAAGAGCCTCCAGGACCTCCAGGTATGGAATACAGGGGACGTTTAAGAAGATATGGCCACACACTGGGGCCCTGAGAAGTGAGAGCTTCATGAAAAAAATCAGGGACCCCAGAGTTCCTTGGAAGCCAAGACTGAAACCAGCATTATGAGTCTCCGGGTCAGAATGAAAGAAGAAGGCCTGCCCCAGTGGGGTCTGTGAATTCCCGGGGGTGATTTCACTCCCCGGGGCTGTCCCAGGCTTGTCCCTGCTACCCCCACCCAGCCTTTCCTGAGGCCTCAAGCCTGCCACCAAGCCCCCAGCTCCTTCTCCCCGCAGGGACCCAAACACAGGCCTCAGGACTCAACACAGCTTTTCCCTCCAACCCCGTTTTCTCTCCCTCAAGGACTCAGCTTTCTGAAGCCCCTCCCAGTTCTAGTTCTATCTTTTTCCTGCATCCTGTCTGGAAGTTAGAAGGAAACAGACCACAGACCTGGTCCCCAAAAGAAATGGAGGCAATAGGTTTTGAGGGGCATGGGGACGGGGTTCAGCCTCCAGGGTCCTACACACAAATCAGTCAGTGGCCCAGAAGACCCCCCTCGGAATCGGAGCAGGGAGGATGGGGAGTGTGAGGGGTATCCTTGATGCTTGTGTGTCCCCAACTTTCCAAATCCCCGCCCCCGCGATGGAGAAGAAACCGAGACAGAAGGTGCAGGGCCCACTACCGCTTCCTCCAGATGAGCTCATGGGTTTCTCCACCAAGGAAGTTTTCCGCTGGTTGAATGATTCTTTCCCCGCCCTCCTCTCGCCCCAGGGACATATAAAGGCAGTT GTTGGCACACCCAgccagcagacgctccctcagcaaggacagcagaggaccagctaagaggga +50. Legend:ATTTATGAAGGCA…TNF-α promoter sequence (-1509bp shown) GAGGCATCTGcFOS binding site (-1277bp bis -1286bp) TaagagggaTNFa gene sequence. [file Image_3.tif]
